# Comparison of patient-controlled analgesia and sedation (PCAS) with remifentanil and propofol versus total intravenous anesthesia (TIVA) with midazolam, fentanyl, and propofol for colonoscopy

**DOI:** 10.1097/MD.0000000000037411

**Published:** 2024-04-12

**Authors:** Hua-Yong Song, Li-Jing Shen, Wen Sun, Lu-Di Zhang, Jian-Guo Liang, Guang-Xin Zhang, Xin-Qing Lu

**Affiliations:** a Department of Anesthesiology, Handan First Hospital of Hebei Province, Handan, Hebei, PR China; b 2nd Gastroenterology Department, Handan First Hospital of Hebei Province, Handan, Hebei, PR China.

**Keywords:** colonoscopy, patient-controlled analgesia and sedation, propofol, remifentanil, total intravenous anesthesia

## Abstract

**Background:**

Colonoscopy is a commonly performed gastroenterological procedure in patients associated with anxiety and pain. Various approaches have been used to provide sedation and analgesia during colonoscopy, including patient-controlled analgesia and sedation (PCAS). This study aims to evaluate the feasibility and efficiency of PCAS administered with propofol and remifentanil for colonoscopy.

**Methods:**

This randomized controlled trial was performed in an authorized and approved endoscopy center. A total of 80 outpatients were recruited for the colonoscopy studies. Patients were randomly allocated into PCAS and total intravenous anesthesia (TIVA) groups. In the PCAS group, the dose of 0.1 ml/kg/min of the mixture was injected after an initial bolus of 3 ml mixture (1 ml containing 3 mg of propofol and 10 μg of remifentanil). Each 1 ml of bolus was delivered with a lockout time of 1 min. In the TIVA group, patients were administered fentanyl 1 μg/kg, midazolam 0.02 mg/kg, and propofol (dosage titrated). Cardiorespiratory parameters and auditory evoked response index were continuously monitored during the procedure. The recovery from anesthesia was assessed using the Aldrete scale and the Observer’s Assessment of Alertness/Sedation Scale. The Visual Analogue Scale was used to assess the satisfaction of patients and endoscopists.

**Results:**

No statistical differences were observed in the Visual Analogue Scale scores of the patients (9.58 vs 9.50) and the endoscopist (9.43 vs 9.30). A significant decline in the mean arterial blood pressure, heart rate, and auditory evoked response index parameters was recorded in the TIVA group (*P* < 0.05). The recovery time was significantly shorter in the PCAS group than in the TIVA group (*P* = 0.00).

**Conclusion:**

The combination of remifentanil and propofol could provide sufficient analgesia, better hemodynamic stability, lighter sedation, and faster recovery in the PCAS group of patients compared with the TIVA group.

## 1. Introduction

Colonoscopy has emerged as one of the most frequently performed procedures in modern gastroenterology in patients associated with anxiety and pain in the gut.^[1]^ During colonoscopy, several drugs and methodologies have been used to achieve sedation and analgesia.^[2–5]^ Among various methodologies, patient-controlled analgesia and sedation (PCAS) is a well-established method that provides safe and effective analgesia and sedation for individual patients according to their requirements.^[6]^ PCAS procedure administered with a combination of propofol and alfentanil has been shown to be acceptable by patients undergoing colonoscopy.^[7,8]^ To this end, remifentanil is a μ-opioid receptor agonist with a similar analgesic effect to that of fentanyl-based derivatives.^[9]^ Owing to its uniquely rapid enzymatic metabolism, remifentanil possesses a pharmacokinetic advantage of a half-life of 3 minutes in clinical situations requiring a predictable termination effect.^[10]^ However, remifentanil is less preferred in outpatients because of its adverse effects, including respiratory depression. The hypnotic and sedative agents of propofol and the opiate remifentanil, which have rapid onset and termination of action, might be ideal choices for PCAS procedures.^[11]^

Considering these attributes, this study aims to use remifentanil and a low dose of propofol in the PCAS approach, compared with total intravenous anesthesia (TIVA) for elective outpatient colonoscopy. Further, we evaluate the feasibility and efficiency of this method without compromising the safety and satisfaction of the patient.

## 2. Patients and methods

The study was conducted in the endoscopy center of Handan First Hospital, Handan, PR China. The consecutive adult patients (n = 80, ASA I and II) scheduled for elective colonoscopy were enrolled. The exclusion criteria for this study were set as follows: (1) patients with severe impairment of cardiopulmonary function; (2) patients with a history of allergy to propofol, midazolam, fentanyl, or remifentanil; (3) patients with a history of large-bowel surgeries; (4) patients with a history of addiction to opiates; (5) patients with psychiatric or emotional diseases; (6) patients with impairment of hearing. Notably, the trial protocol was approved by the Ethical Committee of the Handan First Hospital (Approval number: 2023-L-001). It should be noted that written and signed informed consent was obtained from all patients who were enrolled in this trial. The intervention study was registered in the Clinical Trial Registration (ID: ClinicalTrials.gov Identifier: NCT06175156).

Patients (n = 80) were randomly divided into 2 different groups using the Excel table random sampling method, including the PCAS and the total intravenous anesthesia group (TIVA groups), with 40 in each group.

The PCAS group of patients was treated with 300 mg (30 mL) of propofol (Diprivan, Astra-Zeneca, Caponago, Milano, Italy), 1 mg of remifentanil (Ruijie, Yichang Humanwell, Hubei, China) and 70 mL saline using the patient-controlled syringe pump (Apollo DDB-I-B, Jiangsu, China). The propofol–remifentanil mixture was prepared just before the procedure. In addition, it should be noted that no signs of emulsification of propofol were observed.^[12,13]^ The drug mixture was continuously administered at an infusion rate of 0.1 mL/kg/min after an initial bolus of 3 mL mixture. Then, a bolus of 1 mL was infused in response to a hand-held button pressed by the patient. Each bolus of 1 ml of the mixture delivered contained 3 mg of propofol and 10 μg of remifentanil. The lockout time interval was maintained at 1 minute. As the fastest speed of the pump was adjusted to 99 mL/h, each bolus dose needed about 36 seconds to be delivered into circulation. Notably, the patients were educated on the working flow of the pump before colonoscopy.

In the TIVA group, patients were administered a single dose of 1 μg/kg of fentanyl (Fentanyl, Yichang Humanwell, Hubei, China), 0.02 mg/kg of midazolam (Liyuexi, Nhwa, Jiangsu, China), and 0.8 mg/kg dose of propofol. Further, the administration followed up the bolus increment of 10 mg of propofol to maintain the auditory evoked response index (AAI) within a range of 30 to 40.^[14]^

**Figure 1.**
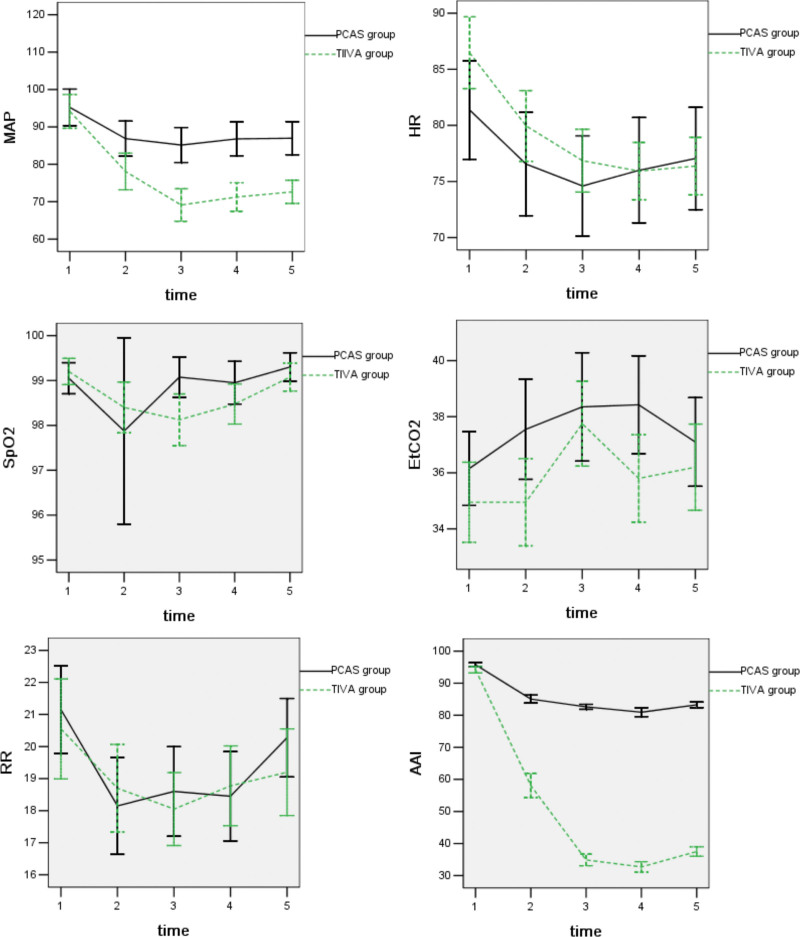
The data show the mean values for arterial pressure (MAP) in beats/min, auditory evoked potential index (AAI), respiratory rate (RR) in the number of respirations per min, end-tidal carbon dioxide (EtCO_2_) in mm Hg, pulse oximetry (SpO_2_) as a percentage, at different stages of colonoscopy. 1: Baseline (3 minutes before administration of the first drug); 2: The time of the start of colonoscopy; 3: The time of colonoscopy inserted to splenic flexure; 4: The time of insertion to cecum; 5: The time of colonoscope withdrawn. Error bar: 95% CI (confidence interval).

All the patients in the 2 recipient groups were subjected to supplemental oxygen through a face mask (FiO_2_ = 40%) during the procedure. Further, the heart rate, pulse oximetry (Spo_2_), end-tidal carbon dioxide (Etco_2_), respiratory rate, and AAI were continuously monitored (PM-9000, Mindray, Shenzhen, China; and A-line AEP Monitor/2, Danmeter, Odense, Denmark). In addition, the noninvasive blood pressure and the Ramsay score were recorded every 2 minutes. Miscellaneous parameters were measured and recorded at 5 different times: 0 referred to as 3 minutes before administration of the first drug; I referred to as the time of the start of colonoscopy; II referred to as the time of insertion of the colonoscope to splenic flexure; III indicated as the time of insertion of the colonoscope to cecum; and IV denoted as the time of withdrawal of colonoscope (Fig. 1).

In the cases of the following adverse events, some critical measures were taken, and the corresponding administrations were executed as follows. Perdipine (0.2–0.4 mg) was administered intravenously in the case of hypertension (MAP higher than 110 mm Hg or 30% of the baseline). The jaw was thrust, and breathing was assisted if necessary in the case of hypoxemia (SpO_2_ < 90%). Ephedrine (6 mg) was administered intravenously in the case of hypotension (MAP lower than 50 mm Hg or 30% of the baseline).^[15]^ Atropine (0.005 mg/kg) was administered intravenously in the case of bradycardia (heart rate lower than 50 bpm). It should be noted that the same experienced endoscopist could perform all these treatment procedures. In addition, all the intravenous anesthesia procedures were executed by the same anesthesiologist except for the PCAS approach. However, the data collection and postoperative statistical analysis were performed by another anesthesiologist who was blinded to the study. Eventually, the recovery of the patient from anesthesia was assessed using the scale of Aldrete and the Observer’s Assessment of Alertness/Sedation Scale (OAA/S).^[16]^ In the case of the OAA/S score reaching 5 points (T1) and the Aldrete score reaching 9 points (T2), the time was measured and recorded from the end of the procedure to the moment. After 30 minutes of the end of the colonoscopy, a 10-cm Visual Analogue Scale (VAS) was recorded to assess the satisfaction of the patients and endoscopist, respectively (left endpoint “completely inadequate sedation” and right endpoint “ideal sedation”). Finally, the adverse events were assessed after 24 to 48 hours of the colonoscopy.

The sample size was determined based on the pre-estimation. During the preliminary test, a *P* < .05 was determined to have a 95% power of 30 patients per group. Further, we sought approval for 40 patients per group to avoid an incomplete study due to severe adverse events and procedures aborted due to bleeding or perforation complications.

The data of the parametric summary statistics were presented as mean and standard deviation (SD). The statistical analyses were performed using the statistical software (Statistical Package for Social Science [SPSS] version 14.0 for Windows, SPSS, Inc, Chicago, IL). The categorical data were analyzed using the Student *t* test and the Pearson Chi-square test with Yates Correction appropriately. The cardiopulmonary parameters were analyzed with repeated measures of multivariate analysis of variance. A *P*-value <.05 was considered statistically significant.

## 3. Results

**Figure 2.**
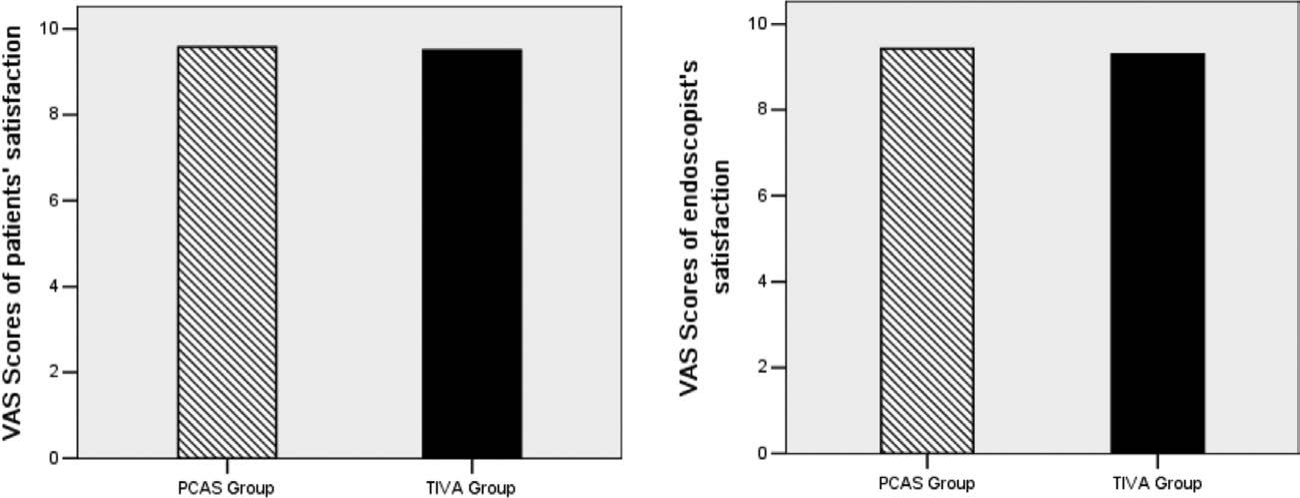
The graphs show the VAS scores of the patients (9.58 vs 9.50) and the endoscopist satisfaction features (9.43 vs 9.30).

Among the total subjects considering the inclusion criteria, a total of 80 patients were found eligible for the study. Further, colonoscopy was performed successfully for all of the recruited subjects. It was observed that no significant differences between the 2 groups (n = 40) were evident concerning age, weight, gender, ASA grade, and the other preliminary basic parameters (Table 1). Further, the adverse events during the procedure are shown in Table 2. Similarly, no significant differences were observed between the 2 groups. The VAS scores assessment for satisfaction in the 2 groups indicated no statistical differences in either of the patient groups (9.58 vs 9.50, *P* = .682) or the endoscopist (9.43 vs 9.30, *P* = .504) (Fig. 2).

**Table 1:** Demographic data and primary clinical parameters.

Parameters	PCAS group (n = 40)	TIVA group (n = 40)	
Male/female	19/21	20/20	
	Mean (SD)		*t* test (*p*)
Age (y)	47.80 (12.82)	51.73 (14.91)	0.21
Weight (kg)	59.13 (11.98)	56.25 (10.05)	0.25
MAP (mm Hg)	95.23 (15.32)	94.15 (14.12)	0.75
HR (n/min)	81.38 (13.74)	86.48 (10.00)	0.06
SpO_2_ (%)	99.05 (1.09)	99.20 (0.91)	0.51
EtCO_2_ (mm Hg)	36.15 (4.12)	34.95 (4.46)	0.22
RR (n/min)	21.15 (4.28)	20.55 (4.87)	0.56
AAI	95.28 (2.02)	94.20 (3.10)	0.07

The mean values of the parameters are shown in (Fig. 1). A decrease of mean arterial blood pressure (MAP), heart rate (HR), and auditory evoked response index (AAI) was observed. Nevertheless, a more significant decline of MAP, HR, and AAI during the procedure was recorded in the TIVA group (*P* < .05). A decrease in SpO_2_ and RR and an increase in EtCO_2_ were noted in both groups without statistical significance (*P* > .05). RR = respiratory rate, SD = standard deviation.

**Table 2:** Adverse events.

Adverse event	PCAS group		TIVA group	
	n	Rate (%)	n	Rate (%)
Hypertension	0	0	0	0
Hypotension	1	2.5	10	25
Hypoxemia	1	2.5	3	7.5
Bradycardia	0	0	5	12.5

**Figure 3.**
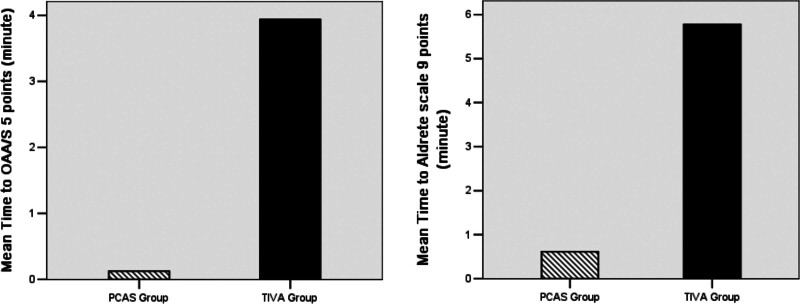
The images show that the mean period from the end of the procedure to the OAA/S scales reached 5 points (0.13 vs 3.94 minutes, *P* = .000), and the period from the end of the procedure to the Aldrete scales reached 9 points (0.61 vs 5.78 minutes, *P* = .000).

Further, the difference in the time interval from the first drug administration to the colonoscope insertion in the 2 groups was not statistically significant (2.71 vs 2.65, *P* = .612). Moreover, the period from the start of the procedure to insertion of the colonoscope to cecum (4.93 vs 5.58 *P* = .339) and to colonoscope withdrawal (7.26 vs 7.91 *P* = .402) were similar in both 2 groups. The period from the end of the procedure to the Aldrete scales reached 9 points, and the OAA/S scales reached 5 points, as shown in Figure 3. It was observed that the PCAS group achieved these standards much faster than the TIVA group, i.e., T1 (0.13 vs 3.94 minutes, *P* = .000) and T2 (0.61 vs 5.78 minutes, *P* = .000). Contrary to the above findings, all the differences between the groups were statistically significant. However, no adverse events were observed in patients in the PCAS group.

## 4. Discussion

Colonoscopy has been considered to be an uncomfortable procedure in scheduled patients who require sedation. Nonetheless, sedation is often associated with the risk of cardiac and respiratory complications, delaying recovery.^[17]^ These complications attributed to sedation may exceed technical complications by a factor of 10.^[18]^ Previous studies indicated that patients would be helped by shorter discharge time and reduced sedation with a patient–control sedation protocol, either with propofol–alfentanil PCS or midazolam–pethidine regimens.^[19]^ In our current study, the PCAS procedure administered with remifentanil and propofol led to a faster recovery time compared to the TIVA approach. Patients in the PCAS group reached the desired recovery scores significantly earlier than those in the TIVA group. Interestingly, both the treatment groups exhibited similar levels of patient and endoscopist satisfaction, including minimal and well-managed adverse events. Together, our findings provided potential benefits of using remifentanil and low-dose propofol for PCAS during colonoscopy, providing efficient sedation, quicker recovery, and patient satisfaction comparable to traditional anesthesia methods.

Among the treatment regimens, the use of a lower dosage of propofol could provide a faster onset of sedation and a more predictable sedative effect compared with the conventional midazolam and meperidine regimen.^[20,21]^ In addition, a lower dosage of propofol could offer a higher level of satisfaction and a shorter period of recovery than the conventional regimen for patients who underwent colonoscopy.^[22]^ Propofol could also act by addressing the limitation of the vomiting effect by the opioids efficiently. Since propofol could not provide sufficient analgesic properties, high concentrations were administered to avoid a response to endoscopy, resulting in possible respiratory depressions (central or obstructive).^[23]^ As a μ-opioid receptor agonist with a similar analgesic effect to that of fentanyl, remifentanil shows respiratory depression and chest wall rigidity. In addition to the significant anesthetic profile, increasing shreds of evidence explained that the propofol–remifentanil combination could be a magnificent pair for spontaneous breathing.^[24]^ Nevertheless, the combination of propofol and remifentanil might result in reduced cardiopulmonary function. Accordingly, the major challenging concern of remifentanil is respiratory depression. In a case, Gold and colleagues indicated that remifentanil could provide adequate analgesia for ambulatory surgery without respiratory depression at an initial dose of 1 μg/kg followed by a continuous infusion of remifentanil at a dose of 0.1 μg/kg/min.^[25]^ In another instance, LaPierre and coworkers demonstrated that the dosages would permit gastroscopic tolerance without resulting in the loss of consciousness or respiratory depression using a sophisticated pharmacodynamic model.^[26]^ According to their findings, this equilibrium could be reached with concentrations of propofol between 1.5 and 2.7 μg/mL, remifentanil up to 0.8 ng/mL, or remifentanil between 3 and 4 ng/mL, and propofol at a dose of 0.6 μg/mL. However, a recently published bi-center retrospective study showed that low-dose remifentanil with propofol reduced the incidence of respiratory depression in gastrointestinal endoscopy compared with remifentanil alone. Motivated by these considerations, this study compared the effect of PCAS with remifentanil and propofol and traditional total intravenous anesthesia. In the previous study,^[27]^ we used lower initial doses of remifentanil of about 0.5 μg/kg and an infusion rate of about 0.1 μg/kg/min. The maximum limit of the total dosage of remifentanil was 0.2 μg kg^−1^ min^−1^. Accordingly, we observed that the onset of respiratory depression was more rapid in the PCAS treatment group than in the TIVA group. Nonetheless, all the patients did not experience significant respiratory depression.

Notably, remifentanil-based patient-controlled analgesia offers superior pain relief compared to other choices, decreasing the need for an epidural and the danger connected with it and reducing the risks with proper management.^[28]^ This study indicated that PCAS with remifentanil and low-dose propofol could provide lighter sedation, a faster recovery time, and a smaller decrease in mean arterial blood pressure compared with that of the TIVA group with fentanyl, midazolam, and propofol in colonoscopy. Although the time interval from the drug administration to the colonoscopy withdrawal was similar,^[29]^ the time from the end of the procedure to the patients’ Aldrete score reached 9 points was significantly shorter in the PCAS group (*P* < .05, 0.61 minutes in the PCAS group vs 5.78 minutes) than in the TIVA group. Notably, an average of 5 minutes was saved for each patient in the PCAS group. Thus, a total of 300 minutes could be saved with the 60 elective colonoscopic cases every day in our center.

Similarly, VAS scores for patient satisfaction were observed in both treatment groups, as reported in the previous studies.^[29]^ Interestingly, several factors, such as young age, psychological distress, pain threshold, higher education, and longer procedure duration, were correlated with the dissatisfaction of the patients in the PCAS group.^[30]^ In this study, no significant statistical difference in patient satisfaction scores was observed between the patient-controlled sedation and the classic intravenous general anesthesia groups. These results could be attributed to the fact that patient-controlled sedation could provide patients with a strong sense of self-control over anxiety, serving to eliminate tension and consequently leading to improved patient satisfaction and a simultaneous reduction in the actual drug requirements. In this context, the most important factor that could be associated with the dissatisfaction of endoscopists in the PCAS group of this study was anismus.^[31]^ In addition, it was challenging to maintain the patient’s privacy during the procedure. Moreover, some patients with more active intestinal distraction reflexes would have a more difficult time entering the scope than they would with the TIVA procedure.^[32]^ In our present work, both the treatment groups of endoscopists showed high satisfaction levels with no statistically significant differences. The satisfaction of endoscopists with the PCAS approach was mainly due to the higher safety profile for patients and the ability to communicate with patients based on changes in their medical conditions compared to the other treatment groups. Several factors affecting endoscopists’ satisfaction included challenges in protecting patient privacy during the procedure and occasional active colonic reflex in some patients. These characteristics would make endoscopists feel that advancing the scope was more challenging than in the general anesthesia group. However, this study found no significant difference in the procedural duration between the 2 groups.

Compared with the standard monitoring and the utilization of pulse oximetry alone, the application of End-tidal carbon dioxide (EtCO_2_) can provide more effective recognition of hypoventilation.^[33]^ In this study, the results demonstrated that a decrease of the SpO_2_ and the respiratory rate could be associated with the increase of the EtCO_2_ in the PCAS group at Stage I. Although the SpO_2_ had already come to the baseline at Stage II, the EtCO_2_ was still at a larger scale, meaning that the depression of the respiratory system still existed at normal SpO_2_ levels. Moreover, the use of EtCO_2_ in non-intubated patients might be limited in terms of its inaccuracy. However, this simple and safe measurement of respiratory monitoring would minimize the inaccuracy as the location of the catheter was in the nostril.

A recent retrospective cohort study involving 259 subjects had received propofol or remifentanil sedation for transcatheter aortic valve replacement.^[34]^ The authors demonstrated the tight linkage of remifentanil use with more hypoxemia and propofol use with a higher rate of vasopressor usage. In the TIVA group, there were 10 patients with hypotension and 3 patients with hypoxemia. In the PCAS group, hypotension was observed in a 74-year-old male patient, and hypoxemia condition was observed in a 73-year-old female patient. Therefore, PCAS might reduce the rate of hypoxemia and hypotension due to the low dose of drugs. Although measures were taken correspondingly and the vital signs were back to the baseline, these events indicated that we might adjust the dosage or the mode of the PCAS for some older people.

The AAI has been programmed as a single numerical value for monitoring the depth of anesthesia.^[35]^ The result of a previous study showed that the AAI could predict the movement of LMA insertion better than the bispectral index.^[14]^ In this study, the AAI was applied to standardize the depth of sedation and anesthesia. Interestingly, no patient movement was found during colonoscopy, while the AAI was maintained between a range of 30 to 40. Furthermore, the electrodes used for the AAI were much cheaper than those used for the bispectral index. Therefore, AAI seemed to be more suitable for monitoring during colonoscopy.

Despite the success in evaluation, a key limitation of this trial included that neither the endoscopist nor the anesthesiologist who performed the sedation and anesthesia were blinded. Considering this aspect, the results could be distorted by their subjective expectations. In the PCAS group, patients could talk with the endoscopist and change their body position in order. Moreover, the sample size of this study was relatively small, requiring a larger sample size to assess the economic cost of the mode. In addition, the participants recruited for this study (only in schools) might have exhibited some bias.

## 5. Conclusion

In summary, this prospective randomized study has demonstrated that the PCAS approach administered with remifentanil and propofol could be safer and more effective compared with TIVA with fentanyl, midazolam, and propofol mixture. In addition, remifentanil mixed with lower dosage propofol might be an ideal choice for the PCAS approach for colonoscopy. We believe that this approach possesses the potential to become an ideal choice for sedation and analgesia during colonoscopy procedures. Furthermore, this method may also be considered an ideal option for other diagnostic and therapeutic procedures that require sedation and analgesia.

## Author contributions

**Conceptualization:** Hua-Yong Song, Li-Jing Shen, Xin-Qing Lu.

**Data curation:** Hua-Yong Song, Wen Sun, Xin-Qing Lu.

**Formal analysis:** Li-Jing Shen, Xin-Qing Lu.

**Investigation:** Xin-Qing Lu.

**Methodology:** Hua-Yong Song, Li-Jing Shen, Lu-Di Zhang, Jian-Guo Liang, Guang-Xin Zhang, Xin-Qing Lu.

**Writing – original draft:** Hua-Yong Song, Li-Jing Shen, Wen Sun, Lu-Di Zhang, Jian-Guo Liang.
